# Associations Between Body Mass Index, Movement Behaviors, Motor Skills, Inhibition and Visuospatial Working Memory in Preschool Children: A Cross-Sectional Study Based on WHO References

**DOI:** 10.3390/children13020306

**Published:** 2026-02-23

**Authors:** Mohamed Amine Ltifi, Kacem Nejah, Fadhel Hammami, Monica Delia Bîcă, Anna Zwierzchowska, Michal Wilk, Dan Iulian Alexe, Mohamed-Souhaiel Chelly

**Affiliations:** 1 Higher Institute of Sport and Physical Education of Gafsa, University of Gafsa, Gafsa 2112, Tunisia; mohamedltifi19@gmail.com; 2 Research Laboratory (LR23JS01) “Sport Performance, Health & Society”, Higher Institute of Sport and Physical Education of Ksar Said, University of Manouba, Tunis 2010, Tunisia; fadhelhammami24@gmail.com (F.H.); mohamedsouhaiel.chelly@issep.uma.tn (M.-S.C.); 3 LR15JS01 EM2S, Education, Motricity, Sport and Health, Higher Institute of Sport and Physical Education, University of Sfax, Sfax 3029, Tunisia; kacemnejah@gmail.com; 4 Faculty of Medical and Behavioral Sciences, “Constantin Brâncuși” University of Târgu-Jiu, 210185 Târgu Jiu, Romania; 5 Department of Physical Education and Adapted Physical Activity, The Jerzy Kukuczka Academy of Physical Education in Katowice, 40-959 Katowice, Poland; a.zwierzchowska@awf.katowice.pl; 6 Institute of Sport Sciences, The Jerzy Kukuczka Academy of Physical Education, 40-065 Katowice, Poland; m.wilk@awf.katowice.pl; 7 Department of Physical and Occupational Therapy, “Vasile Alecsandri” University of Bacău, 600115 Bacău, Romania

**Keywords:** child development, executive functions, motor skills, body mass index (BMI), 24-h movement behaviors, preschool children, anthropometric characteristics

## Abstract

**Highlights:**

**What are the main findings?**

- In Tunisian preschool children aged 4–5 years, Body Mass Index (BMI) according to World Health Organization (WHO) references is primarily associated with anthropometric characteristics such as weight, height, and BMI-for-age z-score.
- Statistically significant associations were observed for age and sleep duration between certain BMI groups, but these differences have limited practical relevance.
- No statistically significant associations were observed between BMI and daily physical activity (PA), sedentary behavior (SB), screen time (ST), executive functions (inhibition, working memory), or gross and fine motor skills (functional mobility, postural steadiness, lower and upper body strength, dexterity).

**What are the implications of the main findings?**

- The small sample size, particularly for the underweight group, limits statistical power, so these results should be interpreted with caution.
- Future studies should include larger and more balanced samples, longitudinal or intervention designs, and examine mediating and moderating factors to clarify the relationships between BMI, PA, and early childhood development.
- Early childhood remains a key window for universal interventions to promote healthy growth, PA, and cognitive-motor development, regardless of weight status.

**Abstract:**

Background: Early childhood represents a key stage for the development of movement behaviors (MB), motor skills (MS), and executive functions (EF). Body Mass Index (BMI), defined according to World Health Organization (WHO) references, may influence these domains early in life. In this context, this cross-sectional observational study aimed to examine the associations between BMI and 24-h MB, MS, and EF in Tunisian preschool children aged 4 to 5 years. Methods: This cross-sectional observational study included 112 Tunisian children aged 4 to 5 years (50 boys, 62 girls), recruited from kindergartens in urban and rural areas. Anthropometric measurements were used to calculate age-specific BMI z-scores and classify children into three BMI categories: below normal, normal, and above normal. Twenty-four-hour MB physical activity (PA), sedentary behavior (SB), and sleep were objectively assessed using accelerometry over five consecutive days. EF (inhibition and working memory) were assessed using standardized cognitive tests, gross MS were evaluated using the Supine Timed Up and Go test (functional mobility), One-Leg Standing Balance test (postural steadiness), Hand Grip Dynamometer (upper body strength), and Standing Long Jump (lower body strength), and fine MS were assessed using the 9-Hole Pegboard Test (dexterity). All tools are validated and standardized for children. Results: Significant differences between BMI categories were observed for anthropometric variables (*p* < 0.05). In contrast, no significant differences were found for 24-h MB, adherence to recommendations, EF, and MS (*p* > 0.05). Only Sleep duration showed a difference significantly between BMI < normal and BMI > normal (*p* = 0.022). Conclusions: In Tunisian preschool children, weight status is primarily associated with differences in physical growth, with no marked relationship to MB, EF, or MS. These findings highlight the importance of universal preventive interventions, particularly focusing on growth monitoring, starting in early childhood. These results should be interpreted with caution and highlight the need for further studies on larger populations to better understand the relationships between BMI, PA, and development in young children.

## 1. Introduction

Early childhood (3–6 years) represents a critical period for motor, cognitive, and behavioral development, with significant implications for future health and lifestyle habits [1,2,3]. During this phase, fundamental capacities such as basic motor skills (MS), self-regulation, and early cognitive processes develop rapidly, laying the foundation for school readiness and long-term physical health [4]. This literature review was guided by recent international conceptual frameworks and empirical studies focusing on 24-h movement behaviors (MB), motor competence, executive functions (EF), and weight status in preschool children. The concept of Twenty-four-hour MB encompasses physical activity (PA), sedentary time, and sleep, which are considered interdependent and essential for optimal development [5,6,7,8,9]. In preschool children, regular PA is associated with better MS and overall functional development, while excessive sedentary behavior (SB) is linked to increased risk of overweight and reduced motor performance [10,11,12]. Sleep and nap practices also contribute to neurocognitive development and body weight regulation [13,14,15]. Recent studies highlight the importance of considering these three behaviors as an integrated movement paradigm rather than as isolated components. Furthermore, the quality and timing of these behaviors may play a crucial role in shaping neuro-motor outcomes, as suggested by observed relationships between nap practices, executive function, and developmental outcomes in Tunisian childcare centers [16]. However, future studies are needed to further examine and clarify these interactions [17]. However, the relationships between 24-h MB, MS, and early cognitive functions remain complex and may be influenced by multiple factors, including individual characteristics, environmental conditions, and cultural practices [16,18,19].

BMI is a key indicator of weight status in children, defined according to World Health Organization (WHO) references [9,15,20,21]. Deviations from normal BMI, such as underweight or overweight, significantly influence fundamental MS and participation in PA [20,22,23]. Systematic reviews and recent studies have confirmed that weight status plays a determining role in the development of physical fitness and motor competence from preschool to middle childhood [24,25,26]. Motor competence has been identified as a central mediator between weight status and participation in PA. For example, elevated BMI is frequently associated with lower motor proficiency and reduced PA levels [19,27]. Anthropometric characteristics may also create biomechanical and physiological constraints that make certain motor tasks more difficult, potentially affecting motor learning and movement confidence [28]. The anthropometric influence on physical fitness and MS in preschool children has also been documented in international studies [21,29].

Beyond motor aspects, weight status appears to be linked to cognitive development, particularly executive function (EF) such as inhibition, working memory, and cognitive flexibility [30,31,32]. Preschool children with excess weight often demonstrate lower executive function and motor performance compared to their normal-weight peers [13,23]. 24-h MB, including sleep and naps, also influence the development of EF [16,24]. EF appear to be influenced not only by biological maturation but also by daily movement patterns and behavioral routines. Furthermore, early environmental factors, such as stimulation at home and school, parental education, and socioeconomic status, can modulate the impact of weight status and daily behaviors on cognitive and motor outcomes [33,34]. Despite this evidence, few studies have simultaneously examined BMI, 24-h MB, motor competence, and EF in a comprehensive analytical framework, particularly in low- or middle-income countries such as Tunisia [33].

However, existing evidence remains inconsistent, with some studies reporting associations between excess weight and reduced motor or executive function, while others find no significant relationship [35]. These inconsistencies highlight the importance of multidimensional research approaches that consider not only BMI but also daily movement patterns, motor competence, cognitive development, and environmental influences. Moreover, such data are particularly scarce in the Tunisian context, where children’s daily routines, cultural practices, and environmental conditions may differ from those in other populations. In Tunisia, preschool children’s daily routines may differ from those in high-income countries, with variability in access to structured physical education, limited organized sports programs, reliance on informal childcare, and sociocultural practices influencing MB and sleep [13,16,24,33,36]. Beyond this, no previous research has concurrently examined BMI, objectively measured 24-h MB, motor competence, and EF within a single framework, highlighting the relevance of the present study. Overall, the literature suggests interrelated but still insufficiently explored pathways linking weight status, movement behavior s, motor competence, and executive functioning in early childhood.

From a developmental perspective, early neuro-motor development emerges from dynamic interactions between biological factors (such as BMI), daily MB, and neurocognitive processes [1,30]. Within this framework, motor competence is considered a key mediator between MB and cognitive outcomes, while EF are shaped by both biological maturation and behavioral experiences [17,20]. Within this framework, BMI may influence motor and cognitive development directly through biomechanical and physiological constraints, and indirectly through its associations with daily MB and environmental contexts (e.g., family routines, preschool environment, and sociocultural practices [19,22,34].

In this context, the present cross-sectional observational study seeks to investigate the associations of weight status, as determined by BMI according to WHO references, on 24-h MB, motor competence, and EF in Tunisian children aged 4 to 5 years. We hypothesize that BMI is associated with daily MB, motor competence, and EF, and that these associations vary according to BMI categories defined by WHO references. The literature indicates that some associations between BMI and motor or EF are well established and consistent [3,20,37,38], whereas other findings remain contradictory or inconclusive, particularly in preschool-aged children [4,36,39], highlighting the importance of our study. To the best of our knowledge, no previous study has concurrently examined the impact of BMI on these three critical domains of neuro-motor development. To address this, the present study aims not only to describe associations between BMI, daily MB, motor competence, and EF, but also to test explicit hypotheses regarding these relationships, thereby enhancing the inferential value of the analyses. By providing data from a middle-income country, this research contributes to the literature and advances our understanding of the early interactions between weight status, MB, and neuro-motor development in early childhood.

## 2. Materials and Methods

### 2.1. Participants

This observational cross-sectional study included 112 preschool children (50 boys and 62 girls) aged 4 to 5 years (mean age: 4.10 ± 0.58 years; median: 4.13 years). Participants were recruited from five kindergartens located in urban and rural regions of the Greater Tunis area, the capital region of Tunisia: El Manar and El Omrane (urban areas), and Cité Zahrouni and Séjoumi (rural areas).

The selected kindergartens included children aged between 4 and 5 years with parental or legal guardian consent. Children with medical conditions, physical disabilities, developmental disorders, or any other conditions that could influence movement, MS, or EF were excluded from participation. The selection of kindergartens aimed to capture variability in environmental and sociodemographic contexts. Although differences between urban and rural areas are well documented, data from both settings were combined to increase statistical power and provide a sample broadly representative of preschool children across these regions, while minimizing the risk of confounding. The kindergartens were randomly selected from both urban and rural areas in Tunisia to ensure a diverse sample. All eligible children aged 4–5 years within each participating kindergarten were invited to take part in the study. A total of 28 children and their parents declined participation, resulting in an overall participation rate of approximately 81%. While the sample is not fully nationally representative, it reflects the typical sociodemographic characteristics of preschool children in these regions.

### 2.2. Procedure

All measurements were performed within the kindergarten setting by a trained research teacher and two research assistants who followed standardized data collection protocols.

Anthropometric measurements, motor skill assessments, and executive function tasks were administered individually. Accelerometers were distributed to children and worn for five consecutive days during daily activities. The order of assessments was standardized across all kindergartens. On the first day, children were fitted with the accelerometer and anthropometric measurements were performed. On the second day, gross motor skill tests were administered in the following order: functional mobility (Supine Timed Up and Go), postural balance (one-leg standing), upper body strength (handgrip), and lower body strength (standing long jump). On the third day, fine motor dexterity (9-Hole Pegboard Test) and the inhibition task (Mr. Ant) were administered. On the fourth day, the working memory task (Go/No-Go) was conducted, and accelerometers were removed at the end of the day, which corresponded to Friday. After each test, children were given sufficient rest while other participants completed their assessments to minimize fatigue and potential learning effects.

Parent and center questionnaires were administered via face-to-face interviews at times convenient for families and kindergarten staff. All questionnaires and instructions were provided in Arabic to ensure clear understanding.

### 2.3. Ethical Approval Statement

All procedures were approved by the Ethics Committee of the Faculty of Medicine of Sousse (CEFMS 121/2022) and were conducted in accordance with the principles of the Declaration of Helsinki. Written informed consent was obtained from parents or legal guardians prior to participation. Children were informed of the study procedures in an age-appropriate manner.

### 2.4. Anthropometry and BMI Classification

#### 2.4.1. Anthropometry

Body height was measured to the nearest 0.1 cm using customizable and repositionable adhesive measuring tapes, following standardized anthropometric procedures. Children stood barefoot in an upright position with the head aligned according to the Frankfurt plane. Each height measurement was performed twice, and when the difference between the two measurements exceeded 0.5 cm, a third measurement was taken, with the mean value retained for analysis.

Body mass was assessed to the nearest 0.1 kg using a calibrated SECA 750 Viva scale (SECA, Hamburg, Germany), with children wearing light clothing and no shoes. Two measurements were obtained, and if the difference exceeded 0.25 kg, a third measurement was conducted and the average value was used.

BMI was calculated as body weight (kg) divided by height squared (m^2^). Height-for-age (HAZ), weight-for-age (WAZ), and BMI-for-age (BAZ) z-scores were calculated using WHO growth reference standards [21].

#### 2.4.2. BMI Classification

According to WHO BMI-for-age z-score classifications [19,38], children were initially categorized as severely thin (<−3 SD), thin (−3 to <−2 SD), normal weight (−2 to +1 SD), at risk of overweight (>+1 to +2 SD), overweight (>+2 to +3 SD), or obese (>+3 SD).

Due to the small number of children in the ‘risk of overweight,’ ‘overweight,’ and ‘obese’ categories, these were combined into a single ‘above-normal BMI’ group to ensure sufficient statistical power for multivariate analyses. We acknowledge that this aggregation may obscure potential heterogeneity within the higher BMI spectrum.

For statistical analyses, categories were regrouped into three BMI groups:

(a) BMI below normal (severe thinness and thinness)
(b) BMI normal
(c) BMI above normal (risk of overweight, overweight, and obesity).

This grouping is consistent with WHO standards for children aged 0–60 months (WHO Anthro) and 61 months to 19 years (WHO AnthroPlus) [15,40], ensuring standardized assessment of weight status adjusted for age and sex.

### 2.5. Physical Activity, Sedentary Behavior, and Sleep

PA, SB, and sleep were objectively measured using ActiGraph GT3X accelerometers (ActiGraph LLC, Pensacola, Florida, USA) attached to the right hip for five consecutive days.

Accelerometers were programmed at a sampling rate of 30 Hz and data were reintegrated into 15-s epochs using a low-frequency filter. ActiLife software (version 6.1.2.1) was used for data processing. A valid day required at least 24 h of data, including a minimum of 6 h of valid waking wear time.

Non-wear time was defined as 20 or more consecutive minutes of zero counts and excluded from analyses. Sleep periods were identified using accelerometer data and confirmed via parental reports and activity logs, and were excluded from PA and SB analyses.

Activity intensity thresholds were defined as follows: SB (<800 counts·min^−1^), light-intensity PA (800–1679 counts·min^−1^), moderate-intensity PA (1680–3367 counts·min^−1^), and vigorous-intensity PA (≥3368 counts·min^−1^) [41,42].

### 2.6. Executive Function (EF)

The assessment of EF was conducted with the Early Years Toolbox [31], focusing on inhibition and visuospatial working memory. Each task lasted approximately 10 min and included a standardized practice phase at the beginning.

The original French versions of the games were translated verbatim into Arabic by a field worker, who was also a physical education teacher, using a forward translation method to ensure accurate adaptation of the content. This Arabic version is currently undergoing validation among Tunisian preschool children [13], but previous studies in this context have confirmed its acceptability and comprehension. The Early Years Toolbox has demonstrated good reliability and validity in international studies with preschool children [13,33]. All executive function assessments were administered individually in a quiet environment within the kindergartens by trained research assistants.

### 2.7. Gross and Fine Motor Skills

MS were assessed using selected tests from the NIH Toolbox [43] and administered in a spacious classroom environment. These assessments have established reliability and validity in international studies of preschool children, supporting their use for evaluating gross and fine MS.

#### 2.7.1. Gross Motor Skills

##### Functional Mobility (Mobility and Posture: Supine-Timed Up and Go)

Functional mobility was assessed using the supine Timed Up and Go test. Children started lying on their back behind a marked line, stood up as quickly as possible, ran to touch a target located 3 m away, and returned to the starting position. One practice trial and two test trials were performed [24,29].

##### Posture: One-Leg Standing Balance Test

Postural stability was assessed using the one-leg standing balance test. Children stood on one leg with their arms alongside the body while the opposite foot was lifted off the ground. The test ended if the child shifted the standing foot or wrapped the free leg around it. Each leg was tested for a maximum of 30 s, and the mean of both trials was calculated for analysis.

##### Upper Body Strength: Hand Grip Dynamometer

Upper body strength was assessed using a hand grip dynamometer (TKK 5825, Grip-A). Children were instructed to squeeze the device with maximum force for at least 3 s without touching their body. One practice trial and two recorded trials were performed for each hand [44].

##### Lower Body Strength and Mobility

Lower limb strength and mobility were assessed via the standing long jump. Participants stood behind a marked line and jumped as far forward as possible using both feet. After a practice jump, two trials were recorded, and the mean distance was used for analysis.

#### 2.7.2. Fine Motor Skills

##### Manipulation: 9-Hole Pegboard Test

Fine motor dexterity was evaluated using the 9-Hole Pegboard Test. Children were asked to place and remove nine pegs one by one as quickly as possible. Timing began when the first peg was touched and ended when the last peg was removed. The total completion time (seconds) was used for analysis. This test has demonstrated good reliability and validity for assessing fine MS in children [45].

### 2.8. Questionnaires

#### Center Information Questionnaire

A Center Information Questionnaire was administered via interview to kindergarten directors. The questionnaire collected information on the total number of children enrolled, participation rates, daily nap schedules, meal provision, food policies, and nutrition practices within the center. In addition, the questionnaire included items adapted from the SUNRISE parent/guardian questionnaire to provide further context, such as children’s daily routines, sleep schedules, time spent in screen activities, time spent sitting or restrained, and eating behaviors, including dietary intake at home outside the kindergarten setting. The SUNRISE questionnaire was translated into the two most common local languages, Arabic and French, and caregivers could choose the language they were most comfortable with. Caregivers were also asked about their interactions with the child during meals, playtime, walks, travel, and bedtime routines. These data were used to provide descriptive contextual information about the kindergarten environment and were not included as covariates in the statistical analyses [29].

### 2.9. Statistical Analysis

No a priori sample size calculation was performed, as this study followed a pragmatic observational cross-sectional design within the SUNRISE framework. The sample size was determined by feasibility and participant availability during the data collection period. Data were analyzed using SPSS Statistics for Windows (version 26.0). Continuous variables are presented as mean ± standard deviation (SD), and categorical variables as number (percentage).

We performed a multivariate analysis of variance (MANOVA) to assess the overall effect of BMI category on all outcome variables simultaneously, followed by univariate analyses for each specific variable. Differences between the three BMI categories were examined using the Kruskal–Wallis test for continuous variables and Pearson’s chi-square tests for categorical variables. When a significant overall effect was observed for continuous variables, pairwise comparisons were conducted and the significance values adjusted using the Bonferroni correction for multiple tests were reported. For Kruskal–Wallis test, epsilon-squared (ε^2^) was calculated as a non-parametric measure of effect size and interpreted according to conventional benchmarks (e.g., for ε^2^: 0.01 = small, 0.06 = medium, 0.14 = large). For chi-square tests, Cramer’s V (V) was computed and was interpreted using Cohen’s [46] benchmarks: 0.10 = small, 0.30 = medium, 0.50 = large. Children were classified into three BMI groups (BMI below normal, BMI normal, and BMI above normal) according to WHO reference standards [21].

Children were classified as meeting the PA guidelines if they accumulated an average of ≥180 min/day of total PA, including ≥60 min/day of moderate-to-vigorous PA, as measured by accelerometry. Accelerometer data were processed using ActiLife software (version 6.1.2.1), with non-wear time (≥20 consecutive minutes of zero counts) excluded and a valid day defined as at least 24 h of data, including a minimum of 6 h of valid waking wear time. Sleep periods were identified from accelerometer data and confirmed via parental reports and activity logs, and were excluded from PA and SB analyses. Compliance with the sedentary screen time (SST) recommendation was defined as ≤60 min/day, and compliance with the sleep duration recommendation was defined as 10–13 h per 24-h period, based on parent-reported data. These definitions are in accordance with the WHO Guidelines on PA, SB and Sleep for Children under 5 Years [40]. While children were recruited from both urban and rural kindergartens, the current analyses did not stratify outcomes by urban–rural status, nor test urban–rural as a potential effect modifier. The level of statistical significance was set at *p* < 0.05.

## 3. Results

Table 1 presents the anthropometric characteristics and MB of preschool children according to BMI categories. Significant differences were observed between BMI groups for most anthropometric variables, including weight, height, BMI, height-for-age (HAZ), weight-for-age (WAZ), and BMI-for-age z-scores (BAZ), indicating differences in overall growth status across groups. Children with BMI > normal generally exhibited higher HAZ, WAZ, and BAZ values, reflecting greater overall growth compared with their peers.

Pairwise comparisons showed significant differences in age between children with BMI < normal and BMI > normal (*p* = 0.034), although the effect size was very small (ε^2^ = 0.053), suggesting limited practical relevance. Sleep duration also differed significantly between BMI < normal and BMI > normal (*p* = 0.022), with a small effect size, indicating that this result should be interpreted with caution.

No significant differences were observed between BMI groups for light, moderate, vigorous, moderate-to-vigorous (MVPA), or total physical activity (TPA), SB, or screen sedentary time, and effect sizes were generally small.

It should be noted that the very small size of the underweight group (n = 5) and the modest size of the above-normal BMI group may reduce statistical power and the ability to detect meaningful differences. Consequently, the absence of significant differences in most movement behavior variables does not necessarily imply the absence of true associations, but rather reflects the detectable effect sizes within the present sample.

Table 2 presents the number and proportion of preschool children meeting the 24-h movement guidelines according to BMI categories.

The majority of children (76.4%) met the guideline of ≥60 min/day of moderate-to-vigorous physical activity (MVPA), with no significant differences between BMI groups (*p* = 0.586, Cramer’s V = 0.111), indicating a small effect size.

Fewer children met the total physical activity (TPA) guideline of ≥80 min/day (40.5%), with non-significant differences across BMI categories (*p* = 0.076, Cramer’s V = 0.239).

Similarly, the combined guideline of ≥60 min/day MVPA and ≥180 min/day TPA was met by 40.4% of children, again with no significant differences between BMI groups (*p* = 0.076, Cramer’s V = 0.239).

Over half of the children (52.7%) met the screen time guideline of ≤60 min/day, with no significant differences (*p* = 0.886, Cramer’s V = 0.046), and most children (81.3%) achieved the recommended sleep duration of 10–13 h/day (*p* = 0.640, Cramer’s V = 0.094).

Only 18% of children met all five 24-h movement recommendations, with no significant differences across BMI categories (*p* = 0.428, Cramer’s V = 0.138). However, the small number of children in the underweight group (n = 5) and modest sample sizes in the above-normal BMI group should be considered when interpreting these findings, as they may limit the statistical power to detect meaningful differences.

Table 3 presents the executive function and gross and fine motor skill outcomes of preschool children according to BMI categories.

No significant differences were observed between BMI groups for inhibition (*p* = 0.579–0.873) or working memory (*p* = 0.793–0.900), indicating small effect sizes and suggesting that cognitive performance was similar across BMI categories.

For gross and fine MS, no significant differences were detected between groups for functional mobility (*p* = 0.968–0.970), postural steadiness (*p* = 0.435–0.621), lower body strength (*p* = 0.262–0.806), upper body strength (*p* = 0.099–0.797), or dexterity (*p* = 0.261–1.000).

It should be noted that the very small number of children in the underweight group (n = 5) and the modest sample sizes in the above-normal BMI group may limit statistical power, and small associations may not have been detected. Consequently, conclusions should be interpreted cautiously and considered within the context of detectable effect sizes in the present sample.

## 4. Discussion

The current cross-sectional observational study aimed to examine the influence of BMI, classified according to WHO references, on 24-h MB, motor competence, and EF in Tunisian preschool children aged 4 to 5 years. To the best of our knowledge, this is one of the few studies to simultaneously analyze these three key domains of early neuro-motor development in a middle-income country context. Significant differences between BMI categories were observed for anthropometric variables, while only sleep duration differed significantly between children with below-normal and above-normal BMI. In contrast, no significant associations were found between BMI and 24-h MB, adherence to recommendations, EF, or most components of motor competence, including functional mobility, postural steadiness, lower and upper body strength, and dexterity. These findings suggest that BMI is not significantly associated with cognitive, motor, or movement patterns at this age. However, due to the limited sample sizes in certain BMI categories, these results should be interpreted with caution and may reflect either a true absence of association or be partly explained by limited statistical power and the sensitivity of the measurement tools. In particular, potential ceiling or floor effects, the limited number of EF domains assessed, and the psychometric properties of the Arabic versions could have reduced the ability to detect subtle associations. Therefore, the possibility of a Type II error cannot be excluded.

### 4.1. Anthropometric Differences According to BMI Categories

Significant differences were observed between BMI categories for the majority of anthropometric variables. Children with above-normal BMI had higher body weight, height, BMI, and BMI-for-age z-scores (BAZ) compared to their normal-weight or underweight peers. Height-for-age (HAZ) and weight-for-age (WAZ) z-scores were also higher in children with elevated BMI, reflecting greater overall somatic growth. These anthropometric differences are partly expected due to BMI-based classification and should therefore be interpreted with caution, particularly given the well-recognized limitations of BMI in early childhood, including its inability to distinguish between fat mass and lean mass. These findings may reflect a pattern of accelerated global growth, which could be related to mechanisms such as early adiposity rebound and individual growth variability; however, such interpretations remain speculative [13,15,19].

These results are consistent with previous studies reporting clear anthropometric distinctions between BMI categories during early childhood [11,12,27]. For instance, research has shown that children with elevated BMI not only exhibit higher body weight but also greater linear growth indicators, suggesting that BMI captures overall somatic development rather than exclusively excess adiposity, which may help explain the limited or non-statistically detectable associations observed with motor competence and EF [11,13].

A small but statistically significant difference in age was observed between BMI groups, specifically between children with BMI below normal and those with BMI above normal (*p* = 0.034, ε^2^ = 0.053). However, the very small effect size indicates limited practical relevance, suggesting that age is unlikely to meaningfully influence the observed anthropometric differences. Therefore, variations in height and weight appear to be primarily related to weight status rather than age-related maturation, although age was still considered as a covariate in the analyses of associations between BMI, motor competence, and EF [13,19].

### 4.2. BMI and 24-h Movement Behaviors

In the present study, no significant differences were observed between BMI categories regarding objectively measured PA (light, moderate, vigorous, MVPA, or total physical activity), SB, screen time. Post hoc analyses indicated a significant difference in sleep duration between children with below-normal BMI and those with above-normal BMI, but the effect size was very small, suggesting limited practical relevance. These findings suggest that, in preschool children, BMI-related differences do not yet translate into observable disparities in daily MB.

This observation aligns with previous research indicating that associations between BMI and PA tend to strengthen with age, particularly during primary school and adolescence, when children gain more autonomy in choosing their physical activities [5,6,12,42]. During early childhood, environmental constraints, such as structured kindergarten schedules, guided play, and limited screen exposure, may contribute to a relative homogeneity of MB across BMI groups, independent of weight status [1,4,7].

Moreover, although most children met the individual MVPA (76.4%) and sleep (81.3%) recommendations, only a small proportion (~18%) met all 24-h movement guidelines simultaneously, regardless of BMI category. This supports the notion that MB at this age are more strongly influenced by the educational environment, parental practices, and daily routines than by individual body composition [5,12,13,17,33].

These results also underscore the need to account for environmental and contextual factors when assessing the relationship between weight status and PA in early childhood. Interventions aimed at improving MB at this age may need to focus more on increasing structured opportunities for active play and promoting regular sleep routines, rather than relying solely on BMI stratification [5,34].

### 4.3. Adherence to the 24-h Movement Guidelines

Although a relatively large proportion of children met the individual recommendations for moderate-to-vigorous physical activity (MVPA; 76.4%) and sleep (81.3%), only 18% of participants met all five components of the 24-h movement guidelines. This finding is consistent with previous SUNRISE study data from low- and middle-income countries, which consistently report low adherence to combined guidelines, even when individual behaviors are relatively well met [47,48,49]. These patterns may reflect the structure of preschool environments, parental practices, and cultural norms that promote certain behaviors (e.g., adequate sleep or MVPA) but do not necessarily support a fully integrated daily movement balance.

Notably, adherence rates did not differ significantly across BMI categories. The strength of association between BMI and guideline adherence was very small, as indicated by Cramer’s V values ranging from 0.046 to 0.239. This suggesting that, in preschool-aged children, weight status is not strongly associated with daily MB. Given the cross-sectional design and unadjusted analyses, causal inferences cannot be made. Important covariates, such as accelerometer wear time and potential center-level effects, were not controlled and may have influenced these results. It should also be noted that the use of a binary outcome (compliance vs. non-compliance) may reduce sensitivity to detect differences between groups, and that the study may not have had sufficient statistical power to identify potential group differences. Therefore, the absence of statistically significant differences should not be interpreted as evidence of no effect of BMI on daily MB. This observation supports the idea that early childhood represents a key window of opportunity for universal interventions promoting healthy MB, rather than focusing solely on children with excess weight [12,49,50,51,52]. Furthermore, the overall low adherence highlights the need for comprehensive strategies that address all aspects of 24-h movement, including light PA, SB, and sleep, to optimize motor and cognitive development from an early age [6,7,47,50].

### 4.4. BMI, Executive Functions, and Motor Skills

No significant differences were observed between BMI groups for EF (inhibition and working memory) or for gross and fine MS, including functional mobility, postural steadiness, lower and upper body strength, and dexterity. These findings suggest that, in this sample of 4- to 5-year-old preschool children, BMI was not significantly associated with cognitive performance or assessed MS.

This contrasts with some studies reporting poorer motor and cognitive performance among children with overweight or obesity, where excess adiposity has been linked to lower motor coordination and reduced neuromuscular performance in pediatric samples [20,37]. However, such associations are not always consistent, especially in early childhood [21,28], and may only emerge more clearly as children grow older and behavioral and physiological differences accumulate [20,37].

It should also be noted that assessing EF in preschool-aged children has inherent developmental limitations, including possible ceiling or floor effects, variability in children’s understanding of task instructions, and fluctuations in behavior during testing. The EF assessment tools used in the present study may have had limited sensitivity to detect subtle differences between BMI groups, which should be considered when interpreting these non-significant findings.

While our observational findings generally indicate no significant associations between BMI and EF or MS, prior research shows mixed results. Variations in study design, participant age, cultural context, measurement tools, and analytical approaches likely explain these inconsistencies. Given the observational nature of our study, causal relationships cannot be inferred. Nonetheless, our study provides additional nuance by concurrently examining BMI, objectively measured 24-h MB, MS, and EF in a middle-income country context, using culturally adapted and validated instruments.

Several potential pathways may help explain why BMI might or might not impact motor and cognitive outcomes in preschool children. Physiologically, elevated BMI may create musculoskeletal or postural constraints that could influence motor performance [19,20,53]. Neurocognitively, differences in energy metabolism, nutrition, or early brain development may subtly affect EF [1,2,30,31]. Behaviorally, variations in daily activity patterns, sleep routines, and SB associated with BMI could also modulate developmental outcomes [4,5,12,47]. In our sample, the absence of significant associations may indicate that at this early age, environmental factors such as structured preschool schedules, guided play, and consistent routines outweigh individual differences in BMI, buffering potential effects on EF and MS [3,7,34]. Considering these physiological, neurocognitive, and behavioral mechanisms provides a more nuanced interpretation of our findings and helps contextualize inconsistencies in the literature.

Finally, the very small number of children in the underweight group (n = 5) and the modest sample size in the above-normal BMI group may limit statistical power, and small associations could have gone undetected. These results should therefore be interpreted with caution and do not imply causal effects of BMI on PA, MS, or EF at this age [12,20,28].

### 4.5. Implications for Child Development and Public Health

Although no significant associations were observed between BMI, 24-h MB, EF, or MS in our sample, the preschool period remains a critical window for promoting healthy development. Universal interventions targeting all children, regardless of weight status, are therefore essential [38,39].

In the Tunisian context, these findings suggest several actionable strategies for educators, healthcare providers, and policymakers. Kindergarten programs could incorporate daily structured physical activities, guided play to develop MS, and regular nap or rest routines to support both cognitive and motor development [33], Parents and caregivers could be engaged through community workshops to encourage balanced daily activity, reduce sedentary time, and ensure adequate sleep at home [43,46]. Public health initiatives should also consider local cultural practices, available resources, and urban–rural differences to ensure feasibility and sustainability [54].

By integrating these educational and environmental strategies from early childhood, Tunisian stakeholders can help establish healthy lifestyle habits, potentially prevent overweight, and support optimal motor and cognitive outcomes in young children [47,50,51].

### 4.6. Strengths and Limitations of the Study

This study’s main strengths include the objective assessment of 24-h MB via accelerometry, the use of WHO-standardized BMI classifications, and the thorough evaluation of MS and EF. The inclusion of children from both urban and rural settings enhances the eco-logical validity of the findings and better reflects the diversity of living contexts in Tunisia.

However, several limitations should be acknowledged:

Observational design and sample size: This is an observational study with a convenience sample and no a priori sample size or statistical power calculation, which may limit the generalizability of the results and increase the risk of Type II error. All statistical analyses were based on unadjusted comparisons, and important covariates, such as accelerometer wear time, were not controlled, potentially influencing the results.

Small subgroup size: A key limitation is the very small size of the underweight BMI group (n = 5), which reduces the reliability of between-group comparisons and increases the risk of Type II errors, limiting the ability to detect meaningful differences. This small sample size may also have prevented the detection of subtle associations between underweight status and cognitive, motor, or movement behaviors, highlighting the need for cautious interpretation of null findings. Future studies should aim to recruit larger and more balanced BMI groups to ensure sufficient statistical power for detecting differences across all BMI categories, including underweight children.

BMI category aggregation: Combining the ‘risk of overweight,’ ‘overweight,’ and ‘obese’ categories into a single ‘above-normal BMI’ group is statistically practical due to small subgroup sizes but may obscure heterogeneity within the higher BMI spectrum.

Executive function and motor skill assessment: EF assessment in preschool children has inherent developmental limitations, such as possible ceiling or floor effects, and only two EF domains were assessed, which may limit the ability to detect differences between BMI groups. Importantly, the Arabic version of the Early Years Toolbox (EYT) is still undergoing full psychometric validation in Tunisian preschool children, which means that the sensitivity of the executive function measures may differ from the original fully validated version, potentially limiting the ability to detect subtle differences between BMI groups. This limitation should be considered when interpreting null findings for executive functions, and highlights the need for future studies using fully validated and culturally adapted instruments. While accelerometers capture MB, they may not fully reflect activity quality or intensity. Future research could complement accelerometer data using additional observational tools, parent reports, or structured interviews to provide a more complete assessment.

Other unmeasured factors: Dietary intake was not directly assessed, which could partially explain BMI variability independent of MB. Although we collected detailed descriptive data on the kindergarten environment and parental interactions, including socioeconomic status (SES) and parental education, these variables were not included as covariates due to the small sample size. These unmeasured factors including SES, genetics, family routines, dietary habits, and the broader socioeconomic environment may have influenced BMI, movement behaviors, and neuro-motor development. Future research should incorporate these variables to better understand their contributions to observed outcomes.

Statistical considerations: All analyses were based on the detectable effect sizes within this sample. Small effect sizes and limited power should be considered when interpreting null findings. Overall, these limitations indicate that caution is warranted when interpreting the lack of significant differences in EF, MS, and MB according to BMI. Future studies should employ larger and more balanced BMI groups, use more sensitive and fully validated executive function assessments, and apply refined motor skill measures to better detect subtle associations between BMI and developmental outcomes. Beyond observational designs, future research should incorporate intervention studies (e.g., randomized controlled trials) and explicitly examine potential mediators (such as PA intensity, sleep quality, and dietary patterns) as well as moderators (including socioeconomic status, kindergarten environment, and parental practices), in order to establish causal relationships and better understand the mechanisms underlying the BMI movement motor EF relationship.

## 5. Conclusions

This cross-sectional study indicates that, in Tunisian preschool children aged 4–5 years, BMI according to WHO references is primarily associated with anthropometric characteristics such as weight, height, and BMI-for-age z-score. Small differences were observed in age and sleep duration between certain BMI groups, but their practical relevance appears limited. No significant associations were found between BMI and daily PA (LPA, MPA, VPA, MVPA, TPA), SB or screen time (SB, SST), executive function (inhibition, working memory), or gross and fine MS (functional mobility, postural steadiness, lower and upper body strength, dexterity). Within the detectable effect sizes of the present sample, no statistically significant differences were observed across BMI categories in cognitive, motor, or daily activity behaviors. These results should not be interpreted as evidence of the absence of associations, but rather reflect the limited statistical power related to the very small size of the underweight group. Therefore, these conclusions should be interpreted with caution and highlight the need for further studies on larger samples to better understand the relationships between BMI, PA, and development in young children. In this perspective, strengthening study designs and analytical frameworks is essential to overcome current methodological limitations. Future research should prioritize intervention-based and longitudinal approaches, and explicitly investigate mediating and moderating factors, in order to clarify causal pathways and inform evidence-based strategies for early childhood development.

## Figures and Tables

**Table 1:** Anthropometric characteristics and movement behaviors of preschool children stratified by body mass index (BMI) categories.

Variables	All (n = 112)	BMI < Normal (n = 5)	BMI = Normal (n = 69)	BMI > Normal (n = 38)	*p* Value	Effect Size
					**Kruskal–Wallis test**	
Age (years)	4.10 ± 0.58	3.98 ± 0.56	4.09 ± 0.57	4.07 ± 0.57	1.000 € 0.205 π **0.034 β**	0.053
Weight (kg)	18.6 ± 2.9	17.7 ± 3.1	18.6 ± 2.9	18.6 ± 3.0	**0.005 €** **<0.001 π** **0.001 β**	0.237
Height (cm)	107.4 ± 6.8	105.8 ± 6.8	107.4 ± 6.8	107.1 ± 6.8	**0.007 €** **<0.001 π** **0.001 β**	0.213
Body mass index (BMI)	16.1 ± 2.0	15.7 ± 2.1	16.1 ± 2.0	16.23 ± 2.0	**0.041 €** **<0.001 π** **<0.001 β**	0.719
Height-for-age z-score (HAZ)	0.85 ± 1.20	0.58 ± 1.36	0.85 ± 1.20	0.89 ± 1.22	**0.013 €** **<0.001 π** **<0.001 β**	0.407
Weight-for-age z-score (WAZ)	0.89 ± 1.26	0.75 ± 1.18	0.90 ± 1.26	0.88 ± 1.29	**0.043 €** **0.001 π** **0.007 β**	0.148
BMI-for-age z-score (BAZ)	0.48 ± 1.37	0.19 ± 1.52	0.46 ± 1.37	0.55 ± 1.36	**0.042 €** **<0.001 π** **<0.001 β**	0.722
		**Multivariate analysis of variance (MANOVA)**				
Light PA (LPA, min/day) a	88.3 ± 24.7	79.8 ± 16.8	86.8 ± 24.6	93.3 ± 24.8	0.847 € 0.568 π 0.509 β	
Moderate PA (MPA, min/day) a	61.8 ± 23.8	67.0 ± 24.2	59.9 ± 23.4	64.3 ± 24.0	0.835 € 0.976 π 0.720 β	
Vigorous PA (VPA, min/day) a	18.8 ± 12.8	15.2 ± 10.8	18.9 ± 13.1	19.0 ± 12.3	0.840 € 0.0845 π 0.999β €	
Moderate-to-vigorous PA (MVPA, min/day) a	80.3 ± 34.9	82.2 ± 32.9	78.9 ± 34.9	83.4 ± 34.9	0.982 € 0.998 π 0.854 β	
Total PA (TPA, min/day) a	168.6 ±55.1	162.0 ± 47.8	165.6 ± 56.0	176.7 ± 52.7	0.991 € 0.875 π 0.682 β	
Sedentary behavior (SB, min/day) a	628.6 ± 56.5	653.1 ± 62.0	633.8 ± 57.1	612.2 ± 49.5	0.783 € 0.366 π 0.239 β	
Screen sedentary time (SST, min/day) b	70.8 ± 33.3	72.2 ± 29.2	69.6 ± 32.1	73.1 ± 37.1	0.985 € 0.998 π 0.866 β	
Sleep duration (min/day) b	640.4 ± 53.5	603.2 ± 22.2	641.8 ± 50.8	642.9 ± 59.8	0.266 € **0.022 π** 0.995 β	

Note: Executive function outcomes are presented as raw scores. No age adjustment was applied due to the narrow age range of the sample (3 to >5 years). Abbreviations: BAZ, BMI-for-age z-score; BMI, body mass index; HAZ, height-for-age z-score; LPA, light-intensity physical activity; MPA, moderate-intensity physical activity per day (in minutes); MVPA, moderate-to-vigorous-intensity physical activity; SB, sedentary behavior; SST, sedentary screen time; TPA, total physical activity; VPA, vigorous-intensity physical activity per day (in minutes); WAZ, weight-for-age z-score. Data are presented as mean (95% confidence interval). Bold values indicate statistical significance at *p* < 0.05. Differences were tested using Kruskal–Wallis test and Multivariate analysis of variance (MANOVA). Analytical sample: a n = 89 (BMI < normal (n = 4), BMI = normal (n = 60), BMI > normal (n = 25). b n 112 (BMI < normal (n = 5), BMI = normal (n = 69), BMI > normal (n = 38). €: *p*-value between BMI < normal and BMI = normal; π: *p*-value between BMI < normal and BMI > normal; β: *p*-value between BMI = normal and BMI > normal.

**Table 2:** Number and proportion of preschool children meeting 24-h movement guidelines according to body mass index (BMI) categories.

Variables	Total n (%)	BMI < Normal (%)	BMI = Normal (%)	BMI > Normal (%)	*p* Value	Cramer’s V
≥60 min/day of MVPA, n = 89	68 (76.4%)	4 (80%)	35 (71.4%)	29 (82.9%)	0.586	0.111
≥80 min/day of TPA, n = 89	36 (40.5%)	4 (80%)	16 (32.7%)	16 (45.7%)	0.076	0.239
≥60 min/day of MVPA and ≥180 min/day of TPA, n = 89	36 (40.4%)	4 (80%)	16 (32.7%)	16 (45.7%)	0.076	0.239
≤60 min/day of SST, n = 112	59 (52.7%)	3 (60%)	37 (53.6%)	19 (50%)	0.886	0.046
10–13 h/day of sleep, n = 112	91 (81.3%)	4 (80%)	55 (79.7%)	32 (84.2%)	0.640	0.094
Meeting all 5 recommendations, n = 89	16 (18.0%)	1 (20%)	11 (22.5%)	4 (11.4%)	0.428	0.138

Abbreviations: MVPA, moderate-to-vigorous-intensity physical activity; SST, sedentary screen time; TPA, total physical activity. Note: Analytical sample: n = 89 (BMI < normal (n = 5), BMI = normal (n = 49), BMI > normal (n = 35); n = 112 (BMI < normal (n = 5), BMI = normal (n = 69), BMI > normal (n = 38). Differences between sexes were tested using Pearson chi-square tests.

**Table 3:** Executive function and gross and fine motor skill outcomes of preschool children according to body mass index (BMI) categories (Mean ± SD).

Variables	Total (n = 112)	BMI < Normal (n = 5)	BMI = Normal (n = 69)	BMI > Normal (n = 38)	*p* Value
Inhibition	0.67 ± 0.24	0.57 ± 0.29	0.68 ± 0.24	0.65 ± 0.25	0.579 € 0.727 π 0.873 β
Working memory	1.89 ± 0.74	2.00 ± 1.22	1.85 ± 0.71	1.95 ± 0.72	0.900 € 0.988 π 0.793 β
Functional mobility (s)	5.30 ± 1.41	5.17 ± 1.53	4.84 ± 1.40	4.81 ± 1.43	0.970 € 0.992 π 0.968 β
Postural steadiness (s)	12.6 ± 7.1	13.3 ± 7.8	15.5 ± 9.2	15.0 ± 9.2	0.621 € 0.922 π 0.435 β
Lower body strength (cm)	56.1 ± 20.8	56.6 ± 22.4	57.9 ± 21.5	57.2 ± 21.7	0.806 € 0.411 π 0.262 β
Upper body strength (kg)	7.24 ± 2.52	7.56 ± 3.19	8.21 ± 2.84	8.13 ± 2.87	0.264 € 0.797 π 0.099 β
Dexterity (s)	40.9 ± 9.8	39.4 ± 10.9	36.9 ± 9.9	37.4 ± 10.0	1.000 € 0.793 π 0.261 β

Note: Executive function outcomes are presented as raw scores. No age adjustment was applied due to the narrow age range of the sample (3 to >5 years). €: *p*-value between BMI < normal and BMI = normal; π: *p*-value between BMI < normal and BMI > normal; β: *p*-value between BMI = normal and BMI > normal. Note: Differences were tested using Multivariate analysis of variance (MANOVA).

## Data Availability

The original contributions presented in this study are included in the article. Further inquiries can be directed to the corresponding author.

## Abbreviations

The following abbreviations are used in this manuscript:

Abbreviation	Definition
BMI	Body Mass Index
WHO	World Health Organization
PA	Physical Activity
EF	Executive Functions
MS	Motor Skills
LPA	Light-intensity Physical Activity
MPA	Moderate-intensity Physical Activity
VPA	Vigorous-intensity Physical Activity
MB	Movement Behaviors
MVPA	Moderate-to-Vigorous Physical Activity
TPA	Total Physical Activity
SB	Sedentary Behavior
SST	Sedentary Screen Time
HAZ	Height-for-age z-score
WAZ	Weight-for-age z-score
BAZ	BMI-for-age z-score
